# Implied Spatial Meaning and Visuospatial Bias: Conceptual Processing Influences Processing of Visual Targets and Distractors

**DOI:** 10.1371/journal.pone.0150928

**Published:** 2016-03-08

**Authors:** Davood G. Gozli, Jay Pratt, K. Zoë Martin, Alison L. Chasteen

**Affiliations:** 1 Department of Psychology, University of Toronto, Toronto, Canada; 2 Institute of Psychology, Leiden University, Leiden, the Netherlands; University of Nottingham, UNITED KINGDOM

## Abstract

Concepts with implicit spatial meaning (e.g., "hat", "boots") can bias visual attention in space. This result is typically found in experiments with a single visual target per trial, which can appear at one of two locations (e.g., above vs. below). Furthermore, the interaction is typically found in the form of speeded responses to targets appearing at the compatible location (e.g., faster responses to a target above fixation, after reading "hat"). It has been argued that these concept-space interactions could also result from experimentally-induced associations between the binary set of locations and the conceptual categories with upward and downward meaning. Thus, rather than reflecting a conceptually driven spatial bias, the effect could reflect a benefit for compatible cue-target sequences that occurs only after target onset. We addressed these concerns by going beyond a binary set of locations and employing a search display consisting of four items (above, below, left, and right). Within each search trial, before performing a visual search task, participants performed a conceptual task involving concepts with implicit upward or downward meaning. The search display, in addition to including a target, could also include a salient distractor. Assuming a conceptually driven visual bias, we expected to observe, first, a benefit for target processing at the compatible location and, second, an increase in the cost of the salient distractor. The findings confirmed both predictions, suggesting that concepts do indeed generate a spatial bias. Finally, results from a control experiment, without the conceptual task, suggest the presence of an axis-specific effect, in addition to the location-specific effect, suggesting that concepts might cause both location-specific and axis-specific spatial bias. Taken together, our findings provide additional support for the involvement of spatial processing in conceptual understanding.

## Introduction

There is considerable evidence that spatial symbols can generate attentional bias toward locations in the periphery. One of the first such studies [1] used a variation of the traditional cueing paradigm in which centrally presented arrow cues appeared before the presentation of peripheral targets. Unlike previous studies, the cues were uninformative, and participants were told that the cues did not predict the locations of the upcoming targets. Nevertheless, a cueing effect was observed such that responses to targets at locations compatible with the cue were faster than to targets at incompatible locations. Follow-up experiments confirmed that the effect of arrows was not based on volitional shifts of attention [1,2,3] (for an elaborate treatment of the role of voluntary control, see [4]), or the physical properties of the arrow [5,6]. In addition to arrows, a variety of uninformative symbols have been shown to produce visuospatial bias. These include explicitly directional words (e.g., "left" and "right", [1,7,8]) and words with an implicit spatial meaning. Implicit spatial cues include numbers [9,10], concepts referring to time [11], concepts with a positive or negative valence [12,13,14,15], concepts related to social status and self-esteem [16,17], and concepts referring to divinity and evil [18]. In all these cases, peripheral targets were processed faster at locations compatible with the spatial meaning of the concepts. The standard interpretation of the existing findings is that the conceptually driven spatial bias reveals the robust association between visuospatial mechanisms and conceptual representation [19,20,21,22]. Processing the concepts, accordingly, involves activating the corresponding sensorimotor spatial components.

The spatial bias induced by concepts has, thus far, been found only in variations of the visuospatial cueing paradigm [23]. This paradigm, although elegant and useful, has two important shortcomings. First, it reveals only the effect of the conceptual cues on processing the target. A spatial bias, however, should not only enhance processing of a task-relevant item but also increase the cost of a task-irrelevant distractor. Demonstrating that conceptually driven spatial biases can increase both target facilitation and distractor interference will confirm that the biases reflect not a benefit for compatible cue-target sequences, but a form of visuospatial bias induced by the conceptual cues. Second, in the cueing paradigm target location is often a binary set (e.g., up vs. down), which opens the results to an alternative interpretation that is not based on the intrinsically sensorimotor components of concepts. According to this alternative, it is possible for binary task dimensions (upward/downward conceptual categories, up/down targets) to become artificially linked during the experiment.

The alternative view is based on the logic of *polarity correspondence* [24,25] (see also [26,27,28,29]), which posits the following assumptions. First, any binary task dimension consists of a more dominant value (+polar) and a less dominant value (-polar). For instance, in a two-choice localization task, with above vs. below stimulus locations and left vs. right response locations, the above location might be considered the +polar stimulus value. Similarly, the right-hand response might be considered the +polar response value. Second, being the +polar value causes a feature to more easily map onto (correspond) the +polar value of other task dimensions. Similarly, being the -polar value causes a feature to more easily map onto the -polar value of other task dimensions. Third, the correspondence between features that share common polarity does not require any intrinsic representational overlap between the features. The correspondence is merely due to sharing the same status along their dimension. That is, as a result of the task structure, the concomitant processing of two +polar values (e.g., right and above), or two -polar values, becomes more efficient than the concomitant processing of a +polar value and a -polar value. Applying this reasoning to conceptual cueing studies, one could argue that the effects are not because concepts are intrinsically associated with spatial features, but because of a task-induced correspondence between the conceptual and the spatial polar values [25]. For instance, comprehending positive affective concepts speeds up responding to visual targets above fixation [12], not because of the intrinsically spatial associations between the abstract concepts and space, but because the two happen to share the same status within the given task, i.e., both being the +polar value along their own dimensions.

There has been debate over what empirical demonstration could constitute unique support for either the representational account or the polarity correspondence account [27,30,31,32], and the two accounts are difficult to disentangle as long as the experimental task involves binary dimensions (e.g., up vs. down). Thus, although the spatial biases induced by conceptual cues have been replicated several times, the phenomenon stands on a narrow foundation because of these two issues, which limits both the interpretation and the generalizability of the impact of conceptual processing on spatial bias. To address these limitations, here we extend the effect of implicit spatial cues in another extensively-studied paradigm of visual attention, known as the additional singleton paradigm [33,34]. Importantly, using this paradigm enables us to use a visual task with non-binary target locations.

In this paradigm, participants are typically instructed to identify a visual target (e.g., line orientation) inside a target object that is defined by a relatively subtle feature (e.g., a square placeholder among circles) while attempting to ignore a relatively salient object (e.g., a high-luminance circle) at one of the distractor locations. Because participants search for a unique target feature whose location is unknown, they are susceptible to capture by other unique features in the display [35,36]. Similar to the cueing paradigm, the additional singleton paradigm can provide information on the effect of centrally presented cues on targets appearing at compatible and incompatible locations. The additional singleton paradigm has two important advantages over the cueing paradigms. The first advantage is that target location is not a binary variable. Given that compatibility effects in tasks with binary locations can be interpreted in terms of task-induced association of the task dimensions (e.g., aligning upward cues with the UP target location), instead of conceptually driven spatial bias [28,29], the present method affords a more rigorous test of the association between concepts and spatial codes. It is important to note that, ultimately, we were still interested in comparing visual attention above and below fixation, depending on the compatibility of those locations with a preceding concept. The critical improvement is that our comparison is now in a situation where participants do not attend solely to locations above and below fixation. Thus, although our critical statistical contrast remains between two values, the experimental situation that grounds this contrast does not consist of a binary spatial set. And, indeed, what matters more for the logic of polarity correspondence is experimental task, and not the statistical contrast.

The second advantage is that the paradigm allows testing for two complementary consequences of spatial bias: (a) variation in the speed of target processing, and (b) variation in the amount of interference from the salient distractor. The first consequence has been studied rather extensively, but the second consequence merits some clarification. With regard to the cost of a salient distractor, the received view is that, at least on a subset of trials, attention is involuntarily captured by the salient distractor, before any other item is attended [37]. In the present study, concepts introduce another source of upward or downward spatial bias, which would increase the probability of attending to the concept-compatible location. Consequently, we predict that concepts would increase the number of trials in which attention is initially captured by the salient distractor, which will be reflected by an increased overall cost of distractors at the concept-compatible location. The combination of consequences (i.e., benefit for the target; cost of the salient distractor) makes the additional singleton paradigm ideal for examining the impact of concepts on spatial attention.

## Experiment 1: Cues Present

In the present experiment, a sequence of two words was presented at fixation with the first being a context word (e.g., "COWBOY") and the second being the cue word (e.g., "HAT"). When the context word and the cue word were related, participants were instructed to perform the search task by finding the square placeholder among the three circular placeholders and identifying the slant of the line presented in that placeholder. When the two words were unrelated, participants were instructed to not perform the search task. The conceptual (relatedness judgment) task was used to ensure participants were processing the cue words prior to performing the search task, instead of ignoring the cue words. Building on previous studies [13,26,38,39,40], we only used cues with implicit spatial meaning along the vertical domain (up/down).

On some trials one of the circular placeholders in the search display had a higher salience level than the other stimuli on the screen, which is expected to induce a stimulus-driven spatial bias [37]. We predicted that a cue word would benefit processing at the locations that are compatible with their meaning (e.g., "HAT" followed by an item above fixation). Although cue-target compatibility has been found to also interfere with visual processing [39], the interference often turns into facilitation if (a) there is enough delay between the cue and target presentation [26], (b) when the cues are consciously perceived [41], or (c) when the cue and the target are both featurally and spatially compatible (e.g., perceiving an image of a bird above fixation, after reading the word “BIRD” at fixation; [40]). Because of a relatively long cue-target onset delay and the fact that our task required conscious processing of the words, we predicted that spatial bias brought about by the word cues would facilitate processing at compatible locations. This bias should, in turn, reduce response time (RT) for a cue-compatible target and increase RT for a compatible salient distractor.

### Method

#### Ethics Statement

Participants gave informed written consent to participate in the study in exchange for course credit. All experimental procedures, including the procedures for acquiring consent and post-experiment debriefing, were approved by the Research Ethics Board of the University of Toronto.

#### Participants

Twenty-three University of Toronto undergraduate students participated in this experiment. They all reported normal or corrected-to-normal vision, and were unaware of the purpose of the study.

#### Apparatus and Stimuli

Participants performed the task in dimly lit rooms. Stimuli were presented on 19" CRT monitors set at 1024 × 768 resolution and 85 Hz refresh rate. Using a head/chin-rest, participants' distance from the display was fixed at about 45 cm.

**Fig 1:**
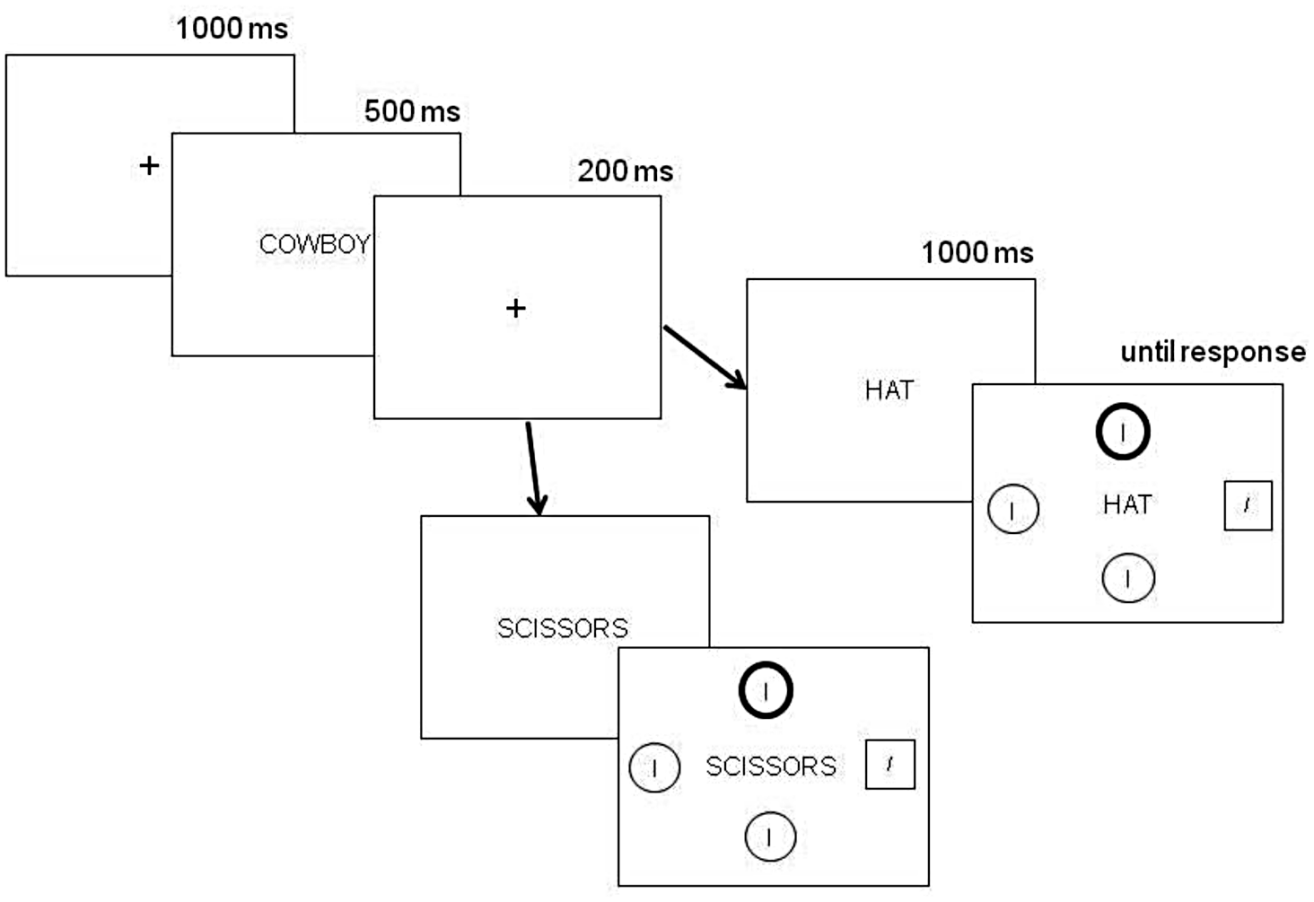
Sequence of events on a sample trial of Experiment 1. The top sequence represents a test trial (context and cue word are related) in which participants were supposed to perform the visual search by identifying the orientation of the tilted line inside the square. The bottom sequence represents a catch trial (context and cue are unrelated) in which participants were supposed to ignore the search display and press the 'z' key.

The display structure and the sequence of events are shown in Fig 1. Stimuli were presented in white against a black background. The words were presented centrally, with each letter subtending approximately.6° × 1.2° of visual angle in size; Arial font). Nine context words were included and each context word was linked to an upward cue, a downward cue, and a catch word. In their respective order (context {upward, downward, catch}), these were RELIGION{GOD, DEVIL, TABLE}, MOOD{HAPPY, SAD, CHALK}, HOUSE{ATTIC, BASEMENT, THUMB}, COWBOY{HAT, BOOT, SCISSORS}, ROOM{CURTAIN, CARPET, BOTTLE}, TREE{BRANCH, ROOT, BOX}, WATER{RAIN, PUDDLE, ZIPPER}, WEIGHT{LIGHT, HEAVY, SHOWER}, ANIMAL{BIRD, WORM, BICYCLE}, and ACTION{FLY, DIG, CLASSROOM}. We used concepts that varied in their categories and in their degree of abstractness, because using cues drawn from a single category in an entire experiment may cause artificial and task-induced mapping between category members and stimulus locations [24,25,26].

The peripheral stimuli (one target and three distractors) were presented above, below, left, and right of the display center (distance from center = 8°). These peripheral stimuli consisted of lines inside placeholders. Distractors consisted of circular placeholders, fit within a 2.4° × 2.4° square (frame width = .04°), and contained a vertical line (length = 1.4°; width = .1°). The target consisted of a square placeholder (2.4° × 2.4°; frame width = .04°) and contained a tilted line ("\" vs. "/"; length = 1.4°; width = .1°). The salient distractor was a circular placeholder with the frame width increased to .24°.

#### Procedure

Each trial began with a central fixation cross ("+") subtending .6° × 6° of visual angle, remaining for 1000 ms. Following fixation, the context word (e.g., "MOOD") was presented for 500 ms. Next, the fixation cross reappeared for another 200 ms and was then replaced by the cue word (e.g., "HAPPY"). Participants were instructed to press the 'z' key if the cue and context words were unrelated (catch trial, e.g., "MOOD" followed by "CHALK"), with these trials serving as catch trials. By contrast, participants were instructed to perform the search task if the two words were related. The search display appeared 1000 ms after the onset of the cue word. Participants were asked to find the tilted line inside the square-shaped placeholder, and press either the left or right arrow key depending on the direction of the target tilt. The cue word and the search display remained on display until a response was recorded or 2000 ms elapsed.

#### Design

Each participant completed 40 practice trials and 480 experimental trials. Out of the 480 trials, 160 trials were catch trials (unrelated context-cue word pairing, e.g., "MOOD" followed by "CHALK") and 320 trials were test trials (related context-cue word pairing). Out of the 320 test trials, 160 trials involved cues with upward spatial meaning (e.g., "MOOD" followed by "HAPPY") and the other 160 trials involved cues with downward spatial meaning (e.g., "MOOD" followed by "SAD"). Participants took self-paced breaks after every 120 trials. Each context word appeared 48 times, and each cue 16 times. The target was equally likely to appear at any of the 4 locations. Therefore, for any given cue word, the visual search target was 25% probable to be at the compatible location, 25% likely to be at the incompatible location, and 50% likely to be at one of the two neutral locations (i.e., along the horizontal axis). On 25% of trials no salient distractor was presented. On the remaining 75% of trials, the salient distractor was equally likely to occupy any of the distractor locations. The target was equally likely to have a left- or rightward tilt and target identity varied independently of target location and distractor location. For the purpose of analysis, we categorized trials based on the cue-target relationship (compatible, incompatible, and neutral), and based on the cue-distractor relationship (compatible, incompatible, neutral, and absent). The term "neutral" refers to left/right peripheral locations.

### Results & Discussion

Three participants were excluded for having percent errors (PEs) above 20% on catch trials. The mean catch- and test-trial PEs of the remaining participants were, respectively, 8% (SE = 1%) and 4% (SE = .6%), indicating that these participants were, for the most part, processing the words. Data were analyzed in two steps, once as a function of cue-target relationship and once as a function of cue-distractor relationship. This is because the location of the salient distractor did not vary independently of the target (e.g., they could not both be compatible or incompatible) and, therefore, the two cannot be treated as independent factors. In analyzing RTs we discarded responses faster than 100 ms and those that fell 2.5 *SD* standard deviations above or below the total mean (3% of trials).

#### Cue-target relationship

**Fig 2:**
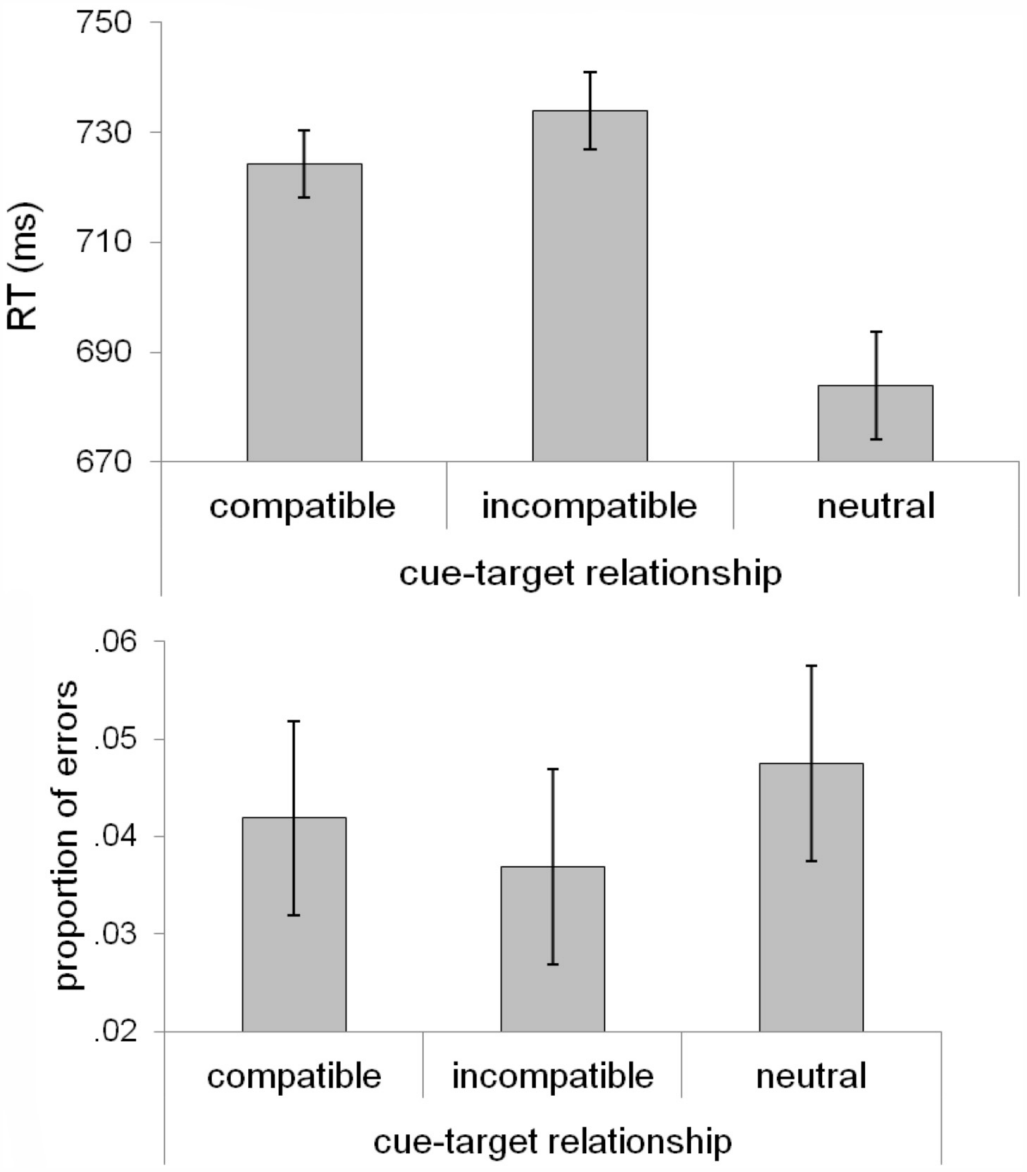
Response time and error data from Experiment 1, graphed as a function of cue-target relationship. Error bars represent 95% within-subject confidence intervals [42].

Mean RTs from the correct test trials were submitted to a one-way repeated measures ANOVA with cue-target relationship (compatible, incompatible, neutral) as the independent factor (Fig 2). This analysis revealed a significant effect (F[2,38] = 29.58, p < .001, ŋ_p_^2^ = .61), which was primarily driven by the faster responses on neutral trials (M ± SE = 684 ± 22 ms) compared to compatible trials (M ± SE = 724 ± 21 ms, t[19] = 5.36, p < .001) and incompatible trials (M± SE = 736 ± 21 ms, t[19] = 6.14, p < .001). More importantly, the comparison between compatible and incompatible trials revealed an advantage for cue-compatible targets (t[19] = 2.18, p < .05, Cohen's d = .49). Responses were faster for targets appearing at the cue-compatible location, compared to the cue-incompatible location.

The same analysis was conducted on error rates (Fig 2), which did not reveal a significant effect (F[2, 38] = 2.53, p = .093, ŋ_p_^2^ = .12). We directly compared error rates across cue-target compatible and incompatible conditions. This comparison did not yield a significant difference (t[19] < 1, p = .40). Therefore, the RT benefit for compatible targets cannot be attributed to a speed-accuracy trade-off.

#### Cue-distractor relationship

**Fig 3:**
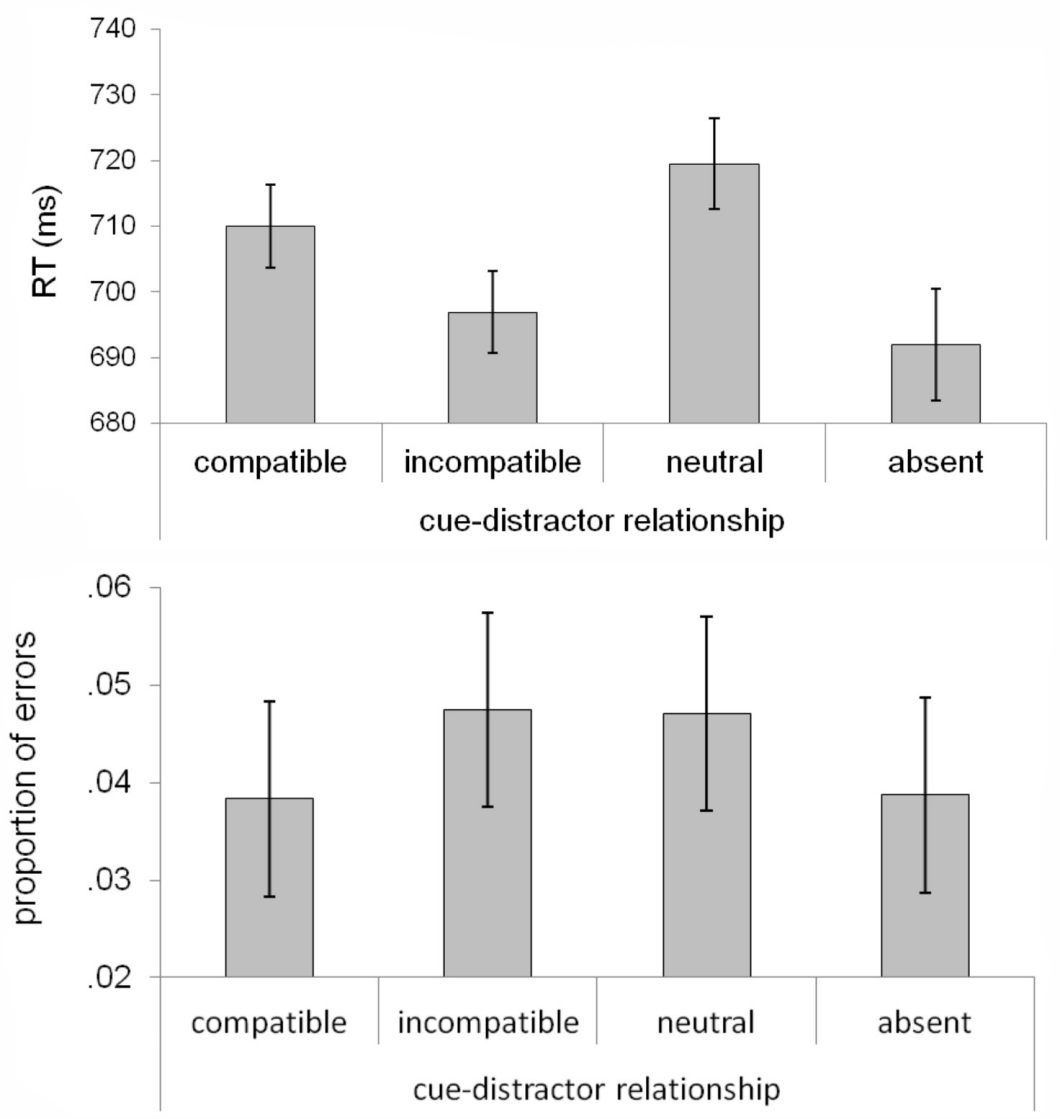
Response time and error data from Experiment 1, graphed as a function of cue-distractor relationship. Error bars represent 95% within-subject confidence intervals.

We submitted the RT data to a similar repeated measures ANOVA, this time with cue-distractor relationship as the factor (compatible, incompatible, neutral, and absent; Fig 3). This analysis revealed a significant effect (F[3,57] = 5.95, p < .01, ŋ_p_^2^ = .24). Responses were faster in the absence of the salient distractor (692 ± 19 ms), compared to in the presence of the distractor (709 ± 22 ms, t[19] = 2.91, p < .01). Furthermore, responses were slower with the distractor on the horizontal axis (i.e., left or right) compared to the distractor on the vertical axis (t[19] = 5.98, p < .001). When considering the vertical axis, the cost of the salient distractor was larger when the distractor appeared at the cue-compatible location (710 ± 22 ms) compared to when it appeared at the cue-incompatible location (697 ± 22 ms, t[19] = 2.96, p < .01). This observation is consistent with a cue-induced spatial bias that not only benefits target processing at the compatible location, but also increases the cost of a salient distractor.

The same analysis was conducted on error data (Fig 3), which revealed a non-significant effect (F[3, 57] < 1). Compatible and incompatible distractors did not lead to significantly different error rates (t[19] < 1, p = .36). Therefore, the cost of cue-compatible distractors cannot be attributed to a speed-accuracy trade-off.

## Experiment 2: Cues Absent

The results of Experiment 1 confirmed our predictions with regard to the ability of conceptual processing to generate spatial bias. Aside the primary purpose of Experiment 1, we also found the unexpected advantage of horizontal locations over vertical locations. This finding motivated this second experiment. Interestingly, the advantage of the horizontal axis was shown both for target processing (i.e., faster responses for targets) and distractor processing (i.e., slower responses for distractors). It is possible that the concepts had an overall interference effect with both locations along the vertical axis. Axis-based, as opposed to location-specific, interference has been reported in previous studies, albeit less commonly [43,44]. To test the possibility of an axis-based effect, we conducted a control experiment in which participants performed a visual search task using identical display features, without the cue and context words. The purpose of this experiment was to see whether the advantage of the horizontal locations would be observed without the conceptual component of the task.

### Method

#### Participants

Twenty new participants took part in this experiment. They were all unaware of the purpose of the study. They all reported normal or corrected-to-normal vision.

#### Stimuli, apparatus, and procedure

These were identical to Experiment 1, with the following exceptions. First, no context or cue words were presented prior to the search display. Following the presentation of the fixation cross (1000 ms), the search display appeared and remained until a response was recorded or 2000 ms elapsed. Second, no catch trials were included in this experiment. Therefore, on each trial participants only reported the orientation of the line inside the target square. The target and the salient distractor were equally likely to appear at any of the 4 locations. For the purpose of analysis, we categorized trials based on the axis of the target location and the axis of the distractor location (vertical vs. horizontal).

#### Design

Each participant completed 20 practice trials and 224 experimental trials. The target was equally likely to appear at any of the 4 locations. On 25% of trials no salient distractor was presented. On the remaining 75% of trials, the salient distractor was equally likely to occupy any of the distractor locations. The target was equally likely to have a left- or rightward tilt and target identity varied independently of target location and distractor location.

### Results & Discussion

Similar to Experiment 1, we analyzed the data in two steps, once as a function of target location axis and once as a function of distractor location axis. In analyzing RTs we discarded responses faster than 100 ms and those that fell 2.5 *SD* standard deviations above or below the mean (3.1% of trials).

#### Target Axis

**Fig 4:**
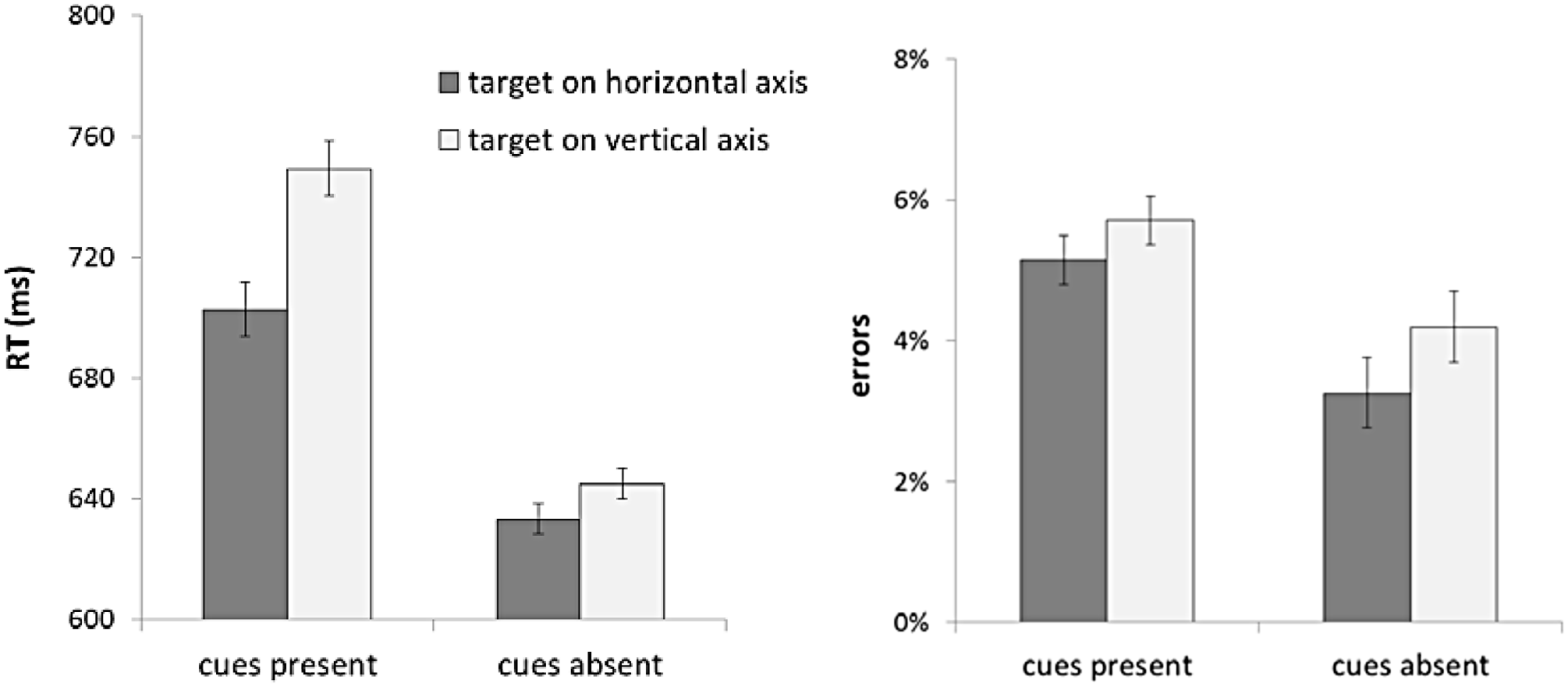
Response time and error data from the two experiments graphed as a function of target axis and cue presence. Error bars represent 95% within-subjects confidence intervals.

Results showed an RT advantage for the horizontal target locations (M ± SE = 633 ± 30 ms) over vertical locations (645 ± 29 ms, t[19] = 2.27, p = .035). Percentage of errors was also consistent with the benefit for targets appearing along the horizontal axis (3.3% ± .6%), compared to the vertical axis (4.2% ± .8%), although the effect on errors did not reach significance (t[19] = 1.81, p = .086). In order to examine the impact of cues, we conducted a mixed ANOVA across the two experiments, using target axis (vertical vs. horizontal) as the within-subjects factor and cue presence (present vs. absent) as the between-subjects factor (Fig 4). An axis-based interference driven by cues would be reflected in a two-way interaction between cue presence and target axis, because the disadvantage of the vertical axis over the horizontal axis would be reduced or eliminated without the conceptual processing component of the task. The analysis revealed a main effect of target axis (F[1,38] = 31.3, p < .001, ŋ_p_^2^ = .45), and a main effect of cue presence (F[1,38] = 5.26, p = .027, ŋ_p_^2^ = .12), as well as a two-way interaction (F[1,38] = 11.06, p < .01, ŋ_p_^2^ = .23). The two-way interaction was driven by a larger effect of axis in the presence of cues (Experiment 1, Cohen's d = 1.15), than in the absence of cues (Experiment 2, Cohen's d = .51). This observation suggests that concepts either facilitated visual selection of items along the horizontal axis or interfered with visual selection of items along the vertical axis. Although both alternatives are logically possible, there is much stronger precedence for the latter possibility in the literature [26,39,40,43,44,45].

Next, error rates were submitted to the same mixed ANOVA (Fig 4). This analysis revealed a main effect of target axis (F[1,38] = 5.63, p = .023, ŋ_p_^2^ = .13), a marginal effect of cue presence (F[1,38] = 3.17, p = .083, ŋ_p_^2^ = .08), but no two-way interaction (F[1,38] < .4, p = .53, ŋ_p_^2^ = .01). The main effect of target axis was driven by the higher error rates for horizontal targets (5% ± .5%) compared to vertical targets (4% ± .5%). The marginal effect of cue presence reflects the higher error rates in the presence of cues (Experiment 1; 5% ± .6%) than in the absence of cues (Experiment 2; 4% ± .6%). Therefore, the RT benefit of the horizontal axis (main effect) seems to be, at least in part, due to a speed-accuracy trade-off. However, the absence of a two-way interaction in error rates suggest that the interaction found in the RT data (larger inter-axis difference in the presence of cues) does not reflect a speed-accuracy trade-off.

**Fig 5:**
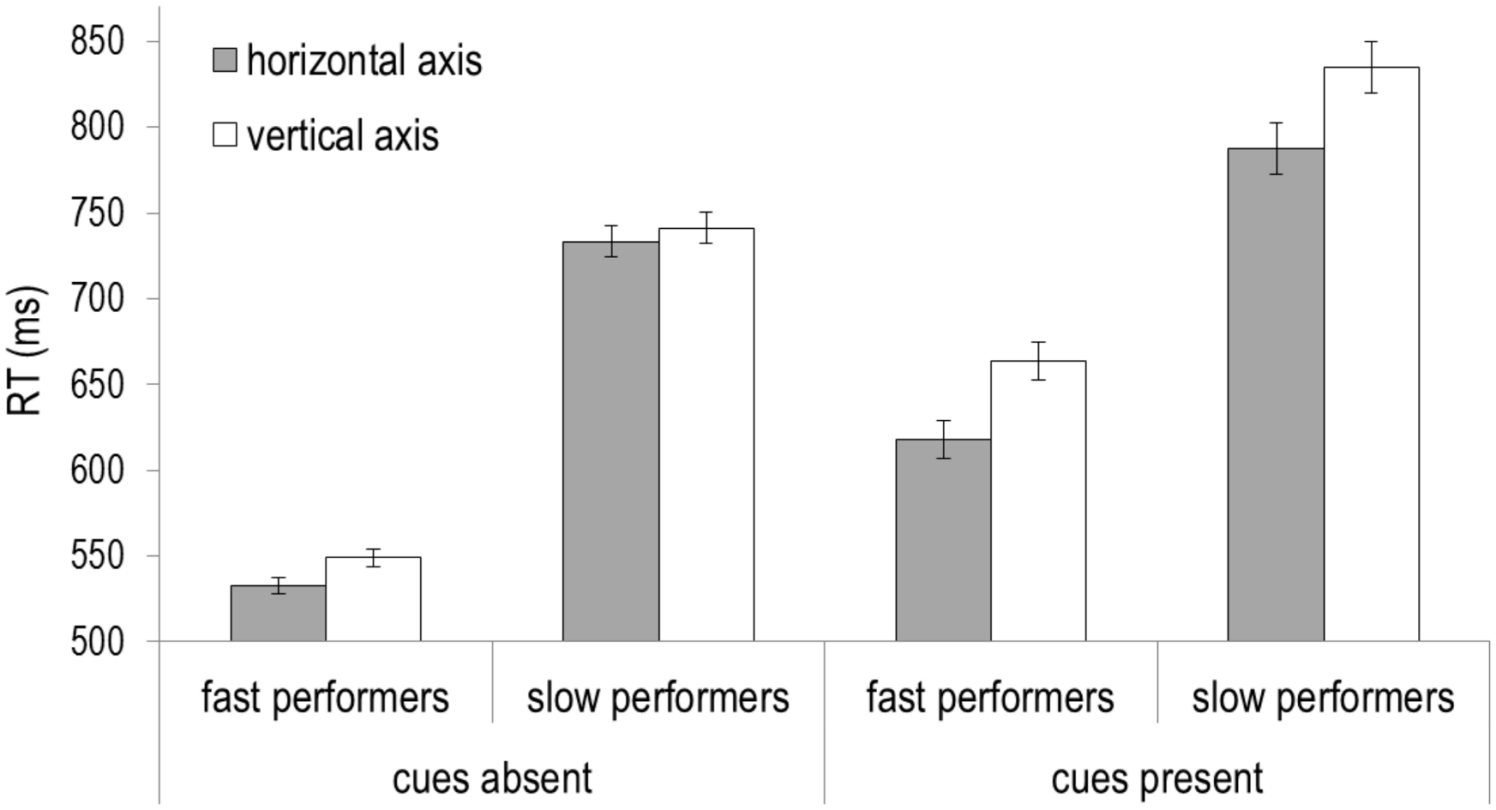
Response time data graphed as a function of cue presence (Experiment 1: cues present vs. Experiment 2: cues absent), performance speed (fast group vs. slow group), and target axis (horizontal vs. vertical). Error bars represent 95% within-subjects confidence intervals. This graph demonstrates that slow performance alone cannot be responsible for the increased advantage of the horizontal axis over the vertical axis.

We should note that performance was faster in the control experiment, in which responses were not contingent on conceptual processing. For this reason, we should consider the possibility that slow performance might be responsible for accentuating the axis effect. To examine the effect of performance speed, we divided participants in both experiments based on their mean RT into two equal groups of fast and slow performers (i.e., a median split), with 10 participants in each subset (see Fig 5). Importantly, the benefit for the horizontal axis was not reduced in faster performers. The axis effect in Experiment 1 (Cohen's d = 1.32 and 1.00, respectively, for fast and slow performers) and Experiment 2 (Cohen's d = .97 and .26, respectively, for fast and slow performers), suggests slow performance was not responsible in increasing the axis effect. Instead, it seems more plausible that conceptual processing generated an inter-axis difference effect in Experiment 1 [43,44].

#### Distractor Axis

As expected, performance was slower in the presence of salient distractors (645 ± 29 ms), relative to without a salient distractor (630 ± 30 ms, t[19] = 2.58, p = .018). However, unlike Experiment 1, the axis of the salient distractor did not significantly modulate its cost in terms of speed of responses (for vertical and horizontal distractor, respectively, 639 ± 30 ms and 645 ± 30 ms, t[19] = 1.2, p = .24). Analysis of errors showed no overall cost of a salient distractor relative to no distractor (error rates with and without the salient distractors, respectively, 3.8% ± .6% and 3.3% ± .8%, t[19] = 1.12, p = .27). However, comparisons of error rates when there was a salient distractor revealed a significantly greater cost of the salient distractor along the vertical axis (4.3% ± .7%) than along the horizontal axis (2.9% ± .7%, t[19] = 2.35, p = .03). Therefore, the axis effect in Experiment 2 might reflect a speed-accuracy trade-off, in contrast to the axis effect found in Experiment 1. This suggest that the presence of the conceptual component in Experiment 1 was crucial for obtaining the axis effect.

**Fig 6:**
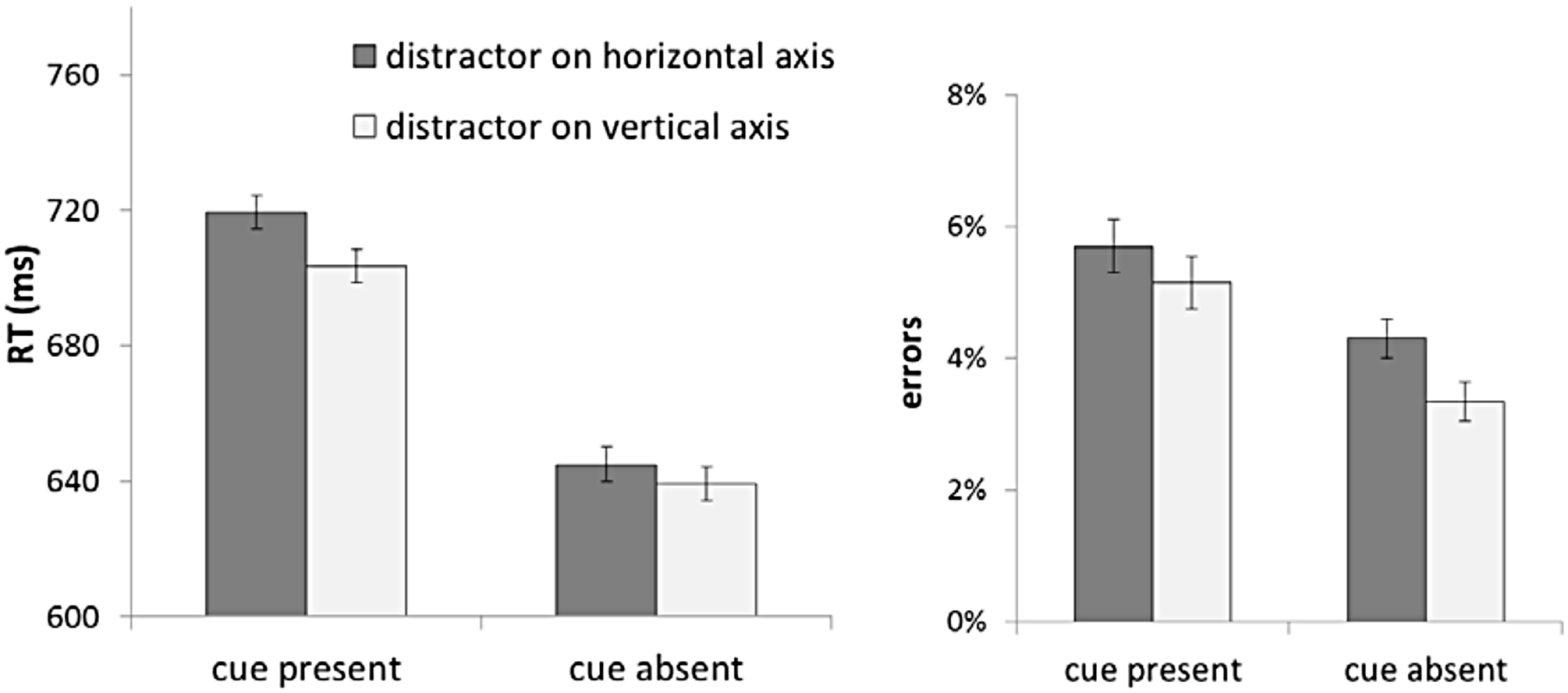
Response time (RT) data and proportion of errors from the two experiments graphed as a function of the salient distractor axis and cue presence. Error bars represent 95% within-subjects confidence intervals.

To examine the axis-specific effect of the concepts on distractor processing, we also conducted a mixed ANOVA across the two experiments, using distractor axis (vertical vs. horizontal) as the within-subjects factor and cue presence (present vs. absent) as the between-subjects factor (Fig 6). An axis x cue interaction could indicate that the concepts, by engaging the vertical spatial dimension, reduced the distractor cost along the vertical axis. Analysis of RTs revealed a main effect of distractor axis (F[1,38] = 9.71, p = .003, ŋ_p_^2^ = .20), a marginal effect of cue presence (F[1,38] = 3.70, p = .062, ŋ_p_^2^ = .09), but no two-way interaction (F[1,38] = 2.12, p = .153, ŋ_p_^2^ = .05). Analysis of error rates, by contrast, revealed no main effect of distractor axis (F[1,38] = 1.83, p = .184, ŋ_p_^2^ = .05), no main effect of cue presence (F[1,38] = 2.68, p = .11, ŋ_p_^2^ = .07), but a significant two-way interaction (F[1,38] = 5.74, p = .022, ŋ_p_^2^ = .13). In light of the absent two-way interaction in RT data, analysis of error data can be interpreted as a conceptually driven difference in processing across the two axes. In the absence of cues, distractor cost was larger for distractors along the vertical axis (M ± SE = 4.3% ± .7%) relative to the horizontal axis (M ± SE = 3.5% ± .6%, t[19] = 2.14, p = .046). In the presence of cues, distractor costs were comparable for distractors along the two axes (M ± SE = 5.3% ± .3% and M ± SE = 5.5% ± .3, for vertical and horizontal distractors, t[19] = .89, p = .38). Together with the effect of cues on target axis (Fig 4), these results are consistent with an interference with processing items along the vertical axis caused by the conceptual cues.

In the preceding analyses, we used cue-target and cue-distractor spatial relationships. This method helps remove any cue-independent advantage for the upper or lower hemifield. In Tables 1 and 2, RT and PE data are presented, divided based on specific target locations. Inspection of these tables reveals a baseline upward and rightward bias in the task. Because we were interested in the effect of congruency between cues and visual items in the present study, and not pre-existing biases toward specific locations, we confined data analysis to factors that reflect cue-target and cue-distractor spatial relationships.

**Table 1:** Mean (SE) response-time data (milliseconds), as a function of cue type, target location, and distractor location.

		Experiment 1		Experiment 2
		upward cue	downward cue	cue absent
target	above	678 (22)	700 (21)	627 (32)
	below	770 (24)	772 (23)	663 (28)
	left	695 (25)	703 (24)	636 (29)
	right	678 (22)	682 (22)	630 (32)
distractor	above	663 (24)	726 (25)	640 (31)
	below	669 (20)	699 (22)	641 (30)
	left	709 (23)	713 (20)	634 (29)
	right	724 (24)	732 (24)	656 (31)
	absent	681 (19)	703 (20)	630 (31)

**Table 2:** Mean (SE) percent-error data presented as a function of cue type, target location, and distractor location.

		Experiment 1		Experiment 2
		upward cue	downward cue	cue absent
target	above	3.9 (.9)	2.5 (.5)	2.4 (.7)
	below	4.9 (.9)	4.5 (1.0)	4.1 (.7)
	left	4.3 (.7)	4.3 (.9)	5.0 (1.1)
	right	4.9 (1.0)	5.6 (1.1)	3.4 (.8)
Distractor	above	5.2 (.9)	5.5 (1.2)	4.6 (.9)
	below	4.0 (1.0)	2.5 (.6)	4.0 (1.0)
	left	4.3 (.9)	4.0 (.9)	3.3 (.6)
	right	4.7 (1.4)	5.8 (1.1)	3.7 (.9)
	absent	4.3 (.7)	3.5 (.8)	3.1 (.8)

## General Discussion

The purpose of this study was to examine the interaction between implicit spatial meaning of concepts and visuospatial processes in the additional singleton paradigm, which included (a) locations along both the horizontal and vertical axes and (b) a salient distractor as well as a search target. Processing concepts, which here served as spatial cues, is thought to automatically activate perceptual features associated with the cue meaning (i.e., simulation, [19]). Conceptually-driven bias in space is commonly interpreted in terms of the underlying representation of the concepts. Namely, it is argued that sensorimotor spatial codes are an essential component of the concepts [26,38,39]. However, tasks with only two possible target locations (above and below) might encourage participants to group together the binary values across those dimensions, which in this case means artificially aligning cue categories and target locations [24]. This interpretation relies on task structure, instead of assuming stable and task-independent associations between conceptual representation and space [13,27,28,29]. Previous studies that used more than two target locations either used only one spatial axis [29] or changed the spatial axis across experimental sessions [17,18]. Our design addressed this limitation by presenting targets at four possible locations within the same experiment.

The second advantage of our method was that it enabled us to observe two distinct consequences of conceptually driven spatial bias. First, we replicated the common finding of faster responses to targets that appear at cue-compatible locations, compared to incompatible locations. Second, we found higher distractor cost when the distractors appeared at the compatible, relative to incompatible, location. To our knowledge, this is the first time that conceptually-driven spatial bias has been demonstrated in terms of an increased cost of distractors appearing at the compatible location. Variations in distractor cost, together with variations in target processing benefit, confirm the role of concepts in biasing spatial attention.

Although we originally aimed to assess the influence of concepts at specific locations along the vertical axis (up vs. down), results from Experiment 1 found the unexpected overall disadvantage at both locations along the vertical axis, relative to locations along the horizontal axis. To test whether conceptual processing might have played a role in the axis effect, we conducted Experiment 2, in which no cues were presented prior to search trials. Comparing the results from the two experiments revealed an overall disadvantage for the vertical axis. Specifically, cue presence increased RT for targets along the vertical axis. The interaction between cue presence and target axis suggests that the cues, by engaging the vertical spatial dimension, might have rendered the vertical axis less available for the visual search task. With regard to distractor processing, in the absence of cues, distractors along the vertical axis were more costly than the horizontal distractors. In the presence of cues, this difference disappeared. This finding also suggests that the cues reduced the impact of vertical distractors, without impacting the influence of horizontal distractors, consistent with the idea that the cues selectively engaged the vertical spatial dimension.

One difference between the two spatial axes has to do with the possible stimulus-response compatibility effect between the location of the search target (left vs. right) and the response to the search target (leftward vs. rightward tilt). Although this form of compatibility applies only when targets appear along the horizontal axis, in the context of the entire experiment it does not introduce a major source of difference between the two axes, because there are equal number of trials with stimulus-response compatible and incompatible combinations. As a consequence, any benefit of the compatibility between search target location and the responding key will be offset by the (equally frequent) incompatible combinations.

Axis-specific interference has been previously reported [43,44], and could be explained in at least three ways. First, the involvement of the vertical spatial codes in processing the concept could render those spatial codes less available for the concurrent perceptual task [45,46]. Second, the need to differentiate and keep separate the two subtasks, the conceptual judgment and the visual search, might have inhibited the features that they share in common [47,48]. Third, repeated activation of the vertical spatial features due to the conceptual subtasks might have caused a habituation in cells that respond to those features, increasing the threshold for activating those feature representations within the visual search task [49].

Previous studies have repeatedly found that conceptual processing can systematically bias spatial processing in both perceptual [39] and motor tasks [50]. A common methodological feature of these studies, however, is that the spatial dimension of the perceptual/motor task is typically a binary set. For instance, participants are required to read a word (e.g., "SKY" or "GROUND"), and then perform one of two actions (upward vs. downward keypress/saccade; e.g., [38,41,50,51,52]) or report a visual target that appears in one of two locations (above vs. below fixation; e.g., [18,26,39,53,54]). The binary spatial dimension might influence how concepts are being processed within the experimental task. According to the logic of *polarity correspondence*, within each binary set (e.g., up/down locations), one values is more salient, and this value is referred to as the default, dominant, or +polar value of the binary dimension. In valence, positive is the +polar value, in up/down and left/right spatial dimensions, respectively, up and right are the +polar values. According to polarity correspondence (a) selecting the +polar values is more efficient than selecting the -polar values, and (b) aligning the +polar values across task dimensions further benefits performance [24]. Consistent with the logic of polarity correspondence, the source of concept-space interaction is the +polar conceptual category, e.g., positive concepts in the category of valence [26,27,28,29]. In essence, the structure of the experimental task is thought to lead participants to form associations between conceptual categories and spatial categories of upward and downward, independently of whether there is an inherently spatial component of the concepts.

There is, however, growing evidence suggesting that polarity correspondence cannot be the sole source of concept-space interactions. For instance, although the vertical and the horizontal spatial domain both have polar values, the conceptual metaphors related to the vertical domain (e.g., valence, social status) have been shown to cause visual bias only in tasks involving vertical, but not horizontal, spatial orienting [14,17]. Furthermore, although the polarity correspondence account holds that the concept-space interaction should be driven primarily by the +polar conceptual category, it has been found that if visual targets follow the concept after brief delays (100 ms), both positive and negative concepts can cause a spatial bias [14]. Furthermore, the P200 event-related potential (ERP) was modulated by cue-target compatibility after both positive and negative concepts [15]. These findings support the metaphorical association account, over the polarity correspondence account.

Another attempt to disentangle polarity correspondence and metaphorical association accounts was made, in which the relative salience of responses was manipulated in a two-choice task by changing keyboard eccentricity [55]. It was assumed that the more eccentric response is more salient than the more centrally located response and, therefore, should be mapped onto the more salient conceptual feature (e.g., large numbers in the number categories, positive concepts in valence categories, and future-related concepts in temporal categories [24]). However, the same number-space and time-space compatibility effect was found regardless of response salience, suggesting that task-induced mapping between concepts and locations are not the only source of the compatibility effects [55].

Our approach to the polarity issue consisted of increasing the number of potential locations in which a target could appear, thus breaking up the binary spatial dimension. Our results, therefore, suggest that the conceptually driven spatial bias does not depend on employing a binary spatial set, and support the view that concepts involve inherently perceptual features [19,20,21]. It should be noted, however, that reducing the task-relevance of either the spatial or the conceptual dimension can reduce or eliminate the interaction [13,51,54,56], which suggests that some degree of task-relevance needs to be assigned to both dimensions for the interaction to arise.

It is worth noting that a similar sequence of two words, the first being a context word (e.g., "cowboy") and the second being an implicitly spatial cue (e.g., "hat" or "boots") has been reported to result in an inverse compatibility effects (i.e., slower responses at compatible locations [39]). Importantly, their words were quickly (150–350 ms) followed by a speeded visual identification task, where peripheral targets were presented above or below the fixation followed by masks (Experiments 2 and 3). They reasoned that conceptual processing evoked perceptual simulation of the concept’s referent, and this simulation interfered with target identification. For instance, reading the sequence *cowboy—hat* may lead to a perceptual simulation that (in addition to activating other perceptual features associated with the concept) engages the cognitive spatial representation of *above*. Since that spatial code is engaged in the simulation, processing a visual target in this region of space is slowed. The conditions for when space-concept interaction results in facilitation or interference are not entirely clear. Recently, we found that cue-target onset asynchrony (CTOA) of around 300 ms tends to result in interference, whereas CTOA of around 1000 ms, in an otherwise identical task, tends to result in facilitation [26]. It seems, therefore, that perceptual simulation of non-spatial features might be more transient than the spatial bias, resulting in early interference and late facilitation.

Other studies have found that cue awareness might play a role in the direction of an effect [41,57,58]. Using relatively short CTOAs, they found that masked cues resulted in interference, while unmasked cues resulted in facilitation of compatible responses. Finally, a recent study found a role for cue-target featural similarity [40]. Specifically, it was found that the spatial compatibility effect facilitated performance if the cue and the target match in their underlying representational features (e.g., the word "BIRD" followed by an image of bird above fixation), but the same spatial compatibility interfered with performance when the cue and the target mismatch in features (e.g., the word "HAT" followed by an image of a bird above fixation). Thus, the specifics of the timing and target presentations likely account for the different effects despite the general commonality in the spatial cueing designs.

To summarize, the present study demonstrated that the spatial bias induced by conceptual processing can be observed without relying on a binary spatial task dimension, supporting the generalizability of such findings. In addition, finding facilitation of target processing as well as increased cost of a salient distractor provides converging evidence for the spatial bias account of the effect of symbolic cues. Finally, we found an axis-specific interference effect with the vertical axis that disappeared in the absence of the conceptual task. A remaining issue that requires further study is the differences and similarities between the processes responsible for location-specific and axis-specific interaction effects. Comparing the impact of concepts associated with vertical and horizontal spatial dimensions (as well as comparing both class of concepts that are spatially neutral) also represents a worthwhile avenue for future investigation. In general, examining conceptual processes as a source of perceptual bias can continue to enrich our understanding of both kinds of capacities.

## Supporting Information

S1 Dataset Complete dataset from Experiments 1 and 2. The entire dataset from Experiments 1 and 2 reported in this article are available to download from the following link: http://goo.gl/iR23wx.
(XLSX)
